# Microglial Nrf2 Activation Orchestrates Ferroptosis Inhibition and α-Synuclein Clearance in Parkinson’s Disease

**DOI:** 10.3390/ijms27104579

**Published:** 2026-05-20

**Authors:** Feifan Chen, Yingneng Liang, Wen Li, Yaxin Guo, Hongchun Liu, Meiyu Geng, Ming Liu, Yu Zhang

**Affiliations:** 1 Key Laboratory of Marine Drugs, Ministry of Education, School of Medicine and Pharmacy, Ocean University of China, Qingdao 266003, China; 2 Shandong Laboratory of Yantai Drug Discovery, Bohai Rim Advanced Research Institute for Drug Discovery, Yantai 264117, China; 3 State Key Laboratory of Drug Research, Shanghai Institute of Materia Medica, Chinese Academy of Sciences, Shanghai 201203, China

**Keywords:** Parkinson’s disease, Nrf2, BV2, ferroptosis, α-synuclein, dimethyl fumarate

## Abstract

Parkinson’s disease (PD) is pathologically characterized by the abnormal aggregation of α-synuclein and the progressive loss of dopaminergic neurons, with microglia-mediated neuroinflammation acting as a pivotal driver of pathogenesis. Ferroptosis, an iron-dependent form of regulated cell death, significantly contributes to PD progression. However, the precise mechanisms governing microglial ferroptosis under α-synuclein pathology, particularly the regulatory role of the master antioxidant transcription factor nuclear factor erythroid 2-related factor 2 (Nrf2), remain elusive. Here, we employed an in vitro BV2 microglial model and an in vivo A53T transgenic mouse model to elucidate the regulatory effects and underlying mechanisms of Nrf2 on ferroptosis-associated phenotypes induced by α-synuclein pre-formed fibrils (PFFs). In vitro, PFF treatment significantly downregulated microglial Nrf2 expression, triggering ferroptosis-associated phenotypes characterized by reactive oxygen species (ROS) accumulation, ferrous iron (Fe^2+^) overload, and elevated lipid peroxidation. Genetic knockdown of Nrf2 exacerbated these ferroptosis-associated phenotypes and accelerated α-synuclein aggregation. Conversely, Nrf2 overexpression or pharmacological activation via dimethyl fumarate (DMF) profoundly suppressed α-synuclein pathology and mitigated ferroptosis-associated signatures. In vivo, microglial activation in the substantia nigra of PD mice was accompanied by marked Nrf2 downregulation. Strikingly, microglia-specific Nrf2 overexpression significantly reversed motor and non-motor deficits (including olfactory and locomotor impairments), demonstrating the sufficiency of microglial protection. Furthermore, systemic administration of the Nrf2 activator DMF not only ameliorated motor dysfunction but also concurrently rescued nigral dopaminergic neurons and reduced striatal α-synuclein aggregation. Taken together, our findings identify Nrf2 downregulation-driven microglial ferroptosis-associated phenotypes as a critical pathogenic mechanism, and demonstrate that targeting this pathway in vivo ameliorates motor and non-motor deficits while preserving dopaminergic neurons in PD mice. These findings support further research on Nrf2 activation and DMF as potential therapeutic strategies for PD.

## 1. Introduction

Parkinson’s disease (PD) is an age-related neurodegenerative disorder characterized by the progressive loss of dopaminergic (DA) neurons and the accumulation of Lewy bodies (LBs), which are primarily composed of misfolded α-synuclein [1]. The pathogenesis of PD is highly complex and multifactorial, encompassing α-synuclein dyshomeostasis, mitochondrial dysfunction, oxidative stress, neuroinflammation, and impaired axonal transport [2]. Among these, chronic neuroinflammation driven by microglia is increasingly recognized as a pivotal factor in PD progression. As the primary immune cells of the central nervous system, chronically activated microglia trigger NADPH oxidase and NF-κB signaling, leading to a massive release of pro-inflammatory mediators and the generation of reactive oxygen species (ROS), ultimately driving the apoptotic loss of DA neurons [3,4].

Ferroptosis is a distinct form of non-apoptotic programmed cell death driven by iron-dependent lipid peroxidation and elevated cellular ROS [5]. Unlike classical pathways such as apoptosis or necrosis, the cardinal hallmarks of ferroptosis include lethal lipid peroxidation and the concomitant functional decline of glutathione peroxidase 4 (GPX4) [6]. Emerging evidence heavily implicates ferroptosis in the pathogenesis of PD. During disease progression, iron pathologically accumulates across various brain regions, with a pronounced predilection for the substantia nigra [7]. The presence of unbound, labile iron catalyzes a cascade of deleterious cellular events, notably the Fenton reaction, which generates highly toxic hydroxyl radicals [8]. This amplifies lipid peroxidation, ultimately executing cell death via apoptosis or ferroptosis [9]. Notably, α-synuclein, the major component of LBs, has been shown to interact directly with iron [10,11,12]. This interaction exacerbates α-synuclein unfolding and accelerates its structural transition from an α-helical conformation into β-sheet-rich oligomers, thereby seeding LB formation [13]. These findings position α-synuclein as a putative master regulator of ferroptosis in PD, highlighting a robust pathological convergence between ferroptotic cell death and PD progression.

Nuclear factor erythroid 2-related factor 2 (Nrf2) is a member of the basic leucine zipper transcription factor family and serves as the master regulator of the cellular antioxidant defense system. Nrf2 orchestrates the expression of detoxifying enzymes and antioxidant proteins to protect cells from oxidative insult [14]. Under basal conditions, Nrf2 is sequestered in the cytoplasm by its inhibitor, Kelch-like ECH-associated protein 1 (Keap1), which facilitates its ubiquitination and proteasomal degradation [15]. Upon exposure to cellular stress, specific cysteine residues in Keap1 are modified, leading to a conformational change that releases Nrf2. Stabilized Nrf2 then translocates to the nucleus, where it binds to antioxidant response elements (AREs) and transactivates the expression of its target genes, including those encoding antioxidant proteins [16].

Currently, available pharmacological interventions for PD are largely symptomatic; even levodopa exerts only limited disease-modifying effects in early-onset PD and fails to halt overall disease progression [17]. Consequently, there is an urgent, unmet clinical need to develop neuroprotective therapies capable of delaying or arresting PD pathogenesis. Dimethyl fumarate (DMF), an Nrf2 activator, functions by binding to cysteine residues within Keap1. This disrupts the Keap1–Nrf2 interaction, facilitating Nrf2 nuclear translocation and the subsequent transactivation of target genes via binding to antioxidant response elements (AREs) [18]. DMF has demonstrated broad therapeutic potential in neurodegenerative contexts; it is currently a first-line oral disease-modifying therapy for relapsing-remitting multiple sclerosis (RRMS) [19], and is undergoing Phase II clinical trials for Alzheimer’s disease (AD). Although direct measurements of DMF/MMF brain concentrations in PD animal models are currently lacking, pharmacokinetic data from patients with secondary progressive multiple sclerosis demonstrated that MMF reaches the cerebrospinal fluid at approximately 11% of plasma levels, with a Tmax delay of only 2 h relative to plasma, indicating ready central nervous system penetration [20].

Despite these insights, the precise mechanisms governing microglial ferroptosis under α-synuclein pathology remain poorly defined. Moreover, it remains to be elucidated whether Nrf2 activation can mitigate α-synuclein preformed fibril (PFF)-induced neurotoxicity by directly suppressing microglial ferroptosis. In this study, we utilized an in vitro cell model alongside an in vivo A53T transgenic mouse model to comprehensively investigate the impact and underlying mechanisms of Nrf2 on α-synuclein PFF-induced ferroptosis. Our findings unveil a novel mechanism wherein microglia-specific Nrf2 activation prevents α-synuclein-driven ferroptosis. This highlights the robust neuroprotective role of Nrf2 in PD, thereby providing a compelling theoretical foundation and a promising therapeutic target for clinical intervention.

## 2. Results

### 2.1. α-Synuclein PFFs Reduce BV2 Microglial Cell Viability and Downregulate Nrf2 Expression in a Dose-Dependent Manner

**Figure 1:**
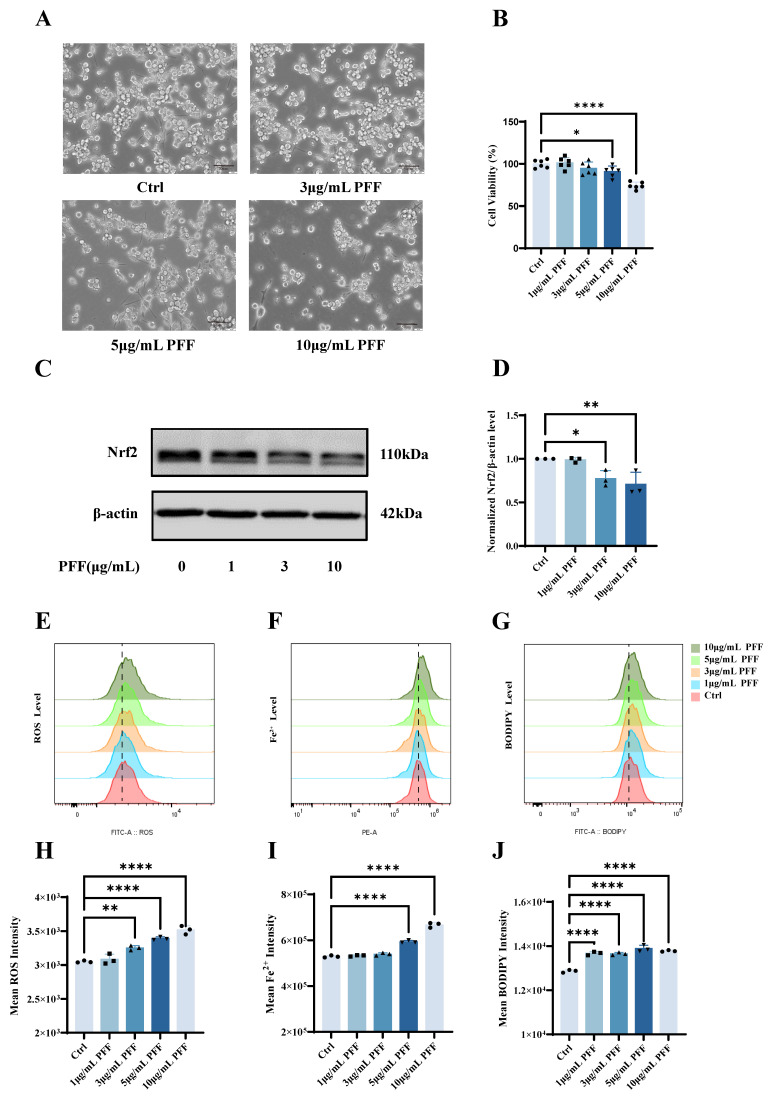
α-Synuclein PFFs induce ferroptosis in BV2 microglia concomitant with Nrf2 downregulation. (**A**) Representative bright-field images of BV2 microglial cells following exposure to increasing concentrations of PFFs for 72 h. Scale bar: 50 μm. (**B**) Quantification of BV-2 cell viability after 72 h treatment with α-synuclein PFFs at the indicated concentrations, as assessed by the CCK-8 assay (*n *= 6). (**C**) Representative Western blot analysis showing changes in the total Nrf2 protein expression in BV2 cells following PFF treatment (*n *= 3). (**D**) Densitometric quantification of total Nrf2 protein levels normalized to the corresponding loading control. (**E**,**H**) Flow cytometric analysis of intracellular ROS levels in BV2 cells using the H2DCFDA probe after α-synuclein PFF exposure (*n *= 3). (**F**,**I**) Intracellular Fe^2+^ levels measured by flow cytometry using the FerroOrange probe (*n *= 3). (**G**,**J**) Assessment of lipid peroxidation in BV2 cells by BODIPY 581/591 C11 staining following PFF treatment (*n *= 3). All experiments were independently repeated at least three times. Data were presented as means ± SD. One-way analysis of variance (ANOVA) followed by Dunnett’s multiple comparison tests in (**B**,**D**,**H**–**J**). * *p* < 0.05, ** *p* < 0.01, and **** *p* < 0.0001.

To investigate the effect of PFFs on BV2 microglial cell viability and explore its potential relationship with ferroptosis and Nrf2 signaling, we first assessed cell survival using the Cell Counting Kit-8 (CCK-8) assay following 72 h of exposure to varying concentrations of PFFs. The results revealed a progressive, dose-dependent decline in cell viability with increasing PFF concentrations (Figure 1A,B). Specifically, treatment with 5 μg/mL and 10 μg/mL PFF reduced cell viability from 100% to 90.87% and 74.19% of the control levels, respectively (*p* = 0.0473 and *p* = 0.0001). These data indicate that PFFs exert a significant cytotoxic effect on BV2 cells in a concentration-dependent manner. We next examined whether PFF exposure alters the protein expression of Nrf2, a master regulator of antioxidant defense. Using whole-cell lysates, we measured the total Nrf2 protein levels via Western blot analysis and found that the total Nrf2 expression was significantly diminished following PFF treatment (Figure 1C,D). Relative to the controls, Nrf2 expression was reduced to 0.7729 ± 0.09264 (*p* = 0.0276) and 0.7073 ± 0.1390 (*p* = 0.0073) in cells treated with 3 μg/mL and 10 μg/mL PFFs, respectively. This downregulation occurred in a clear dose-dependent manner, suggesting that PFF-induced cytotoxicity may involve suppression of the Nrf2 antioxidant pathway.

Ferroptosis is a regulated form of cell death characterized by the iron-dependent accumulation of lipid peroxides. To determine whether the PFF-induced reduction in cell viability is associated with ferroptosis, we evaluated key ferroptotic markers, including intracellular ROS, Fe^2+^, and lipid peroxidation, using flow cytometry. Intracellular ROS levels were measured using the H2DCFDA probe. Consistent with the observed cytotoxicity, PFF treatment led to a dose-dependent increase in ROS production. Compared with the control group, ROS levels rose from 3050 ± 15.95 to 3253 ± 35.02, 3392 ± 23.30, and 3519 ± 62.32 in the 3, 5, and 10 μg/mL PFF groups, respectively (*p* = 0.0015, *p* = 0.0001, *p* = 0.0001), indicating that PFFs induced significant oxidative stress (Figure 1E,H). Similarly, using the FerroOrange probe, we detected a significant accumulation of Fe^2+^ in BV2 cells exposed to increasing concentrations of PFFs. Compared with the controls, Fe^2+^ levels in the 5 and 10 μg/mL PFF groups increased from 529,000 ± 4583 to 593,667 ± 5508 and 667,333 ± 10,786, respectively (*p* = 0.0001 for both) (Figure 1F,I). Furthermore, lipid peroxidation, assessed by the BODIPY™ 581/591 C11 probe, was markedly elevated in PFF-treated cells compared to the controls, with the most pronounced effect observed at the highest PFF concentration. Compared with the control group, lipid peroxidation levels in the 1, 3, 5, and 10 μg/mL PFF groups increased from 12,867 ± 64.82 to 13,673 ± 79.54, 13,654 ± 61.92, 13,892 ± 149.3, and 13,781 ± 34.95, respectively (*p* = 0.0001 for all comparisons) (Figure 1G,J). Taken together, these results demonstrate that PFFs reduce BV2 microglial cell viability in a dose-dependent manner, concomitant with the downregulation of Nrf2 protein expression. The PFF-treated cells also exhibited hallmark features of ferroptosis, including the accumulation of ROS, ferrous iron, and lipid peroxides. These findings collectively suggest that PFF exposure successfully induces ferroptosis-associated phenotypes in BV2 microglial cells, and that this process is closely associated with the suppression of Nrf2 expression.

### 2.2. Nrf2 Silencing Exacerbates α-Synuclein PFF-Induced Ferroptosis in BV2 Microglia

**Figure 2:**
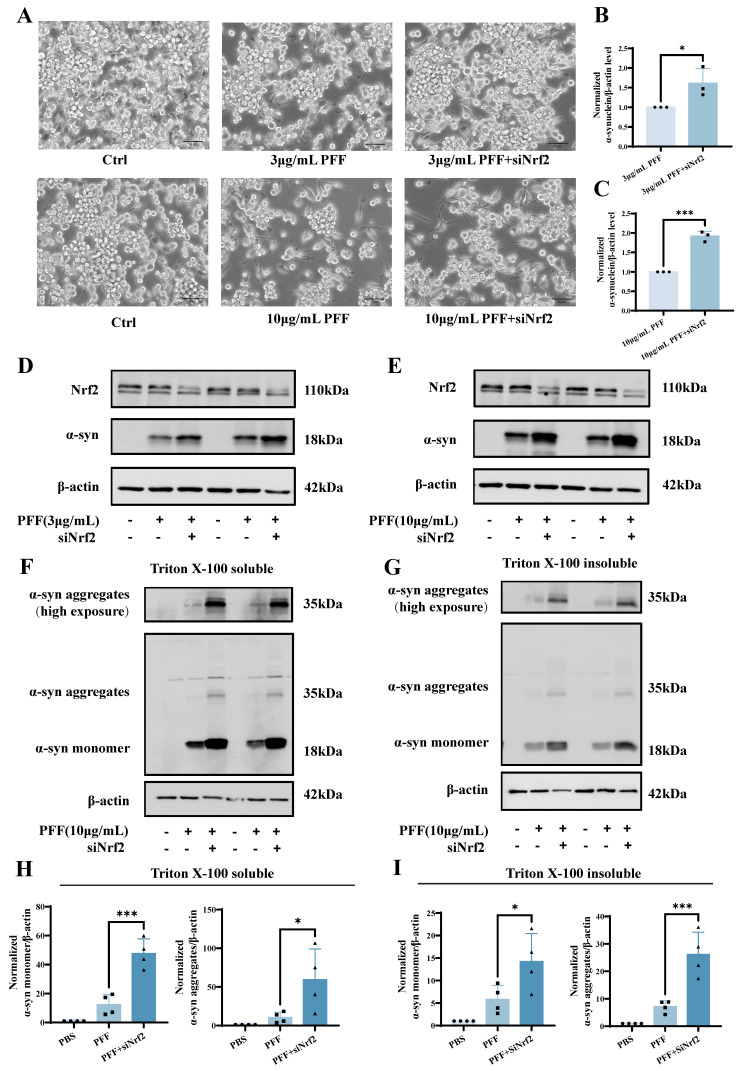
Nrf2 silencing promotes α-synuclein PFF-induced upregulation of intracellular α-synuclein expression. (**A**) Representative bright-field images of BV2 cells transfected with siNrf2 and subsequently treated with 3 or 10 μg/mL PFF (scale bar: 50 μm). (**B**,**C**) Densitometric quantification of α-synuclein protein levels in total cell lysates. (**D**,**E**) Representative Western blot analysis of total Nrf2 and α-synuclein protein expression in BV2 cells following PFF treatment (*n* = 3). (**F**,**G**) Western blot analysis of α-synuclein levels in Triton X-100-soluble and Triton X-100-insoluble fractions (*n* = 4). (**H**,**I**) Quantitative analysis of α-synuclein protein levels in the 1% Triton X-100-soluble and 1% Triton X-100-insoluble fractions. All of the above experiments were carried out more than three biological replicates. All experiments were independently repeated at least three times. Data were presented as means ± SD. Statistical significance was determined by the two-tailed Student’s *t*-test in (**B**,**C**). One-way analysis of variance (ANOVA) followed by Dunnett’s multiple comparison tests in (**H**,**I**). * *p* < 0.05, *** *p* < 0.001.

To examine the impact of Nrf2 depletion on intracellular α-synuclein burden, BV2 microglial cells transfected with control or Nrf2-targeting siRNA were exposed to equivalent doses of PFF. Total α-synuclein levels in whole-cell lysates prepared with RIPA buffer were quantified by Western blotting. Strikingly, Nrf2 knockdown resulted in a marked increase in total α-synuclein levels upon PFF treatment. At 3 μg/mL PFF, α-synuclein protein levels in siNrf2 cells increased to 1.610 ± 0.3762 relative to the PFF-treated control cells (*p* = 0.0484); at 10 μg/mL PFF, this increase reached 1.915 ± 0.1288 (*p* = 0.0003) (Figure 2A–E). These findings indicate that loss of Nrf2 renders microglia more susceptible to either enhanced uptake of exogenous PFF or impaired clearance of intracellular α-synuclein. To determine whether Nrf2 knockdown alters the aggregation state of α-synuclein, Triton X-100 fractionation was performed to separate Triton-soluble and Triton-insoluble fractions from the control and siNrf2-transfected BV2 microglial cells following PFF exposure. The distribution of α-synuclein within each fraction was subsequently analyzed. Nrf2 silencing markedly enhanced PFF-induced α-synuclein accumulation in both soluble and insoluble fractions. Compared with the PFF group, in the soluble fraction, monomeric α-synuclein increased from 12.38 ± 7.034 to 47.71 ± 10.04 (*p* = 0.0001), and aggregated species increased from 10.49 ± 6.859 to 59.43 ± 39.79 (*p* = 0.0284). In the insoluble fraction, monomeric α-synuclein increased from 5.830 ± 3.076 to 14.21 ± 6.225 (*p* = 0.0289), and insoluble aggregate species increased from 7.150 ± 2.149 to 26.15 ± 8.137 (*p* = 0.0007) (Figure 2F–I). Collectively, these findings indicate that Nrf2 deficiency not only elevates the total α-synuclein levels, but more importantly, drives its pathological conversion from soluble monomeric forms to insoluble aggregated species. This underscores a critical role for Nrf2 in maintaining α-synuclein proteostasis in microglial cells.

**Figure 3:**
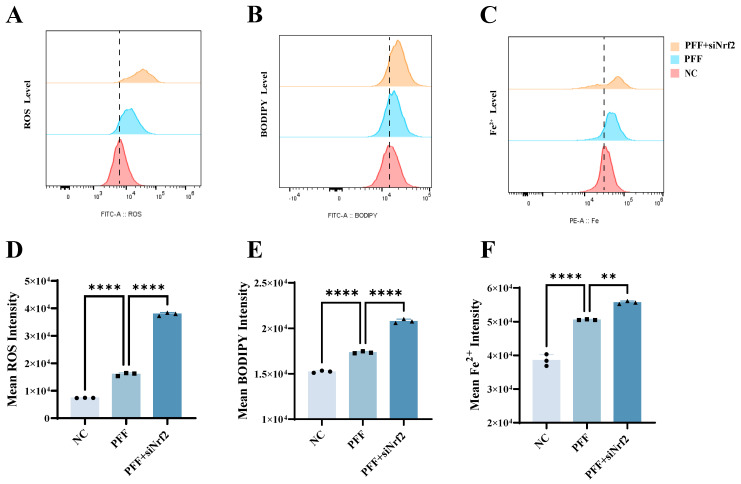
Nrf2 silencing exacerbates α-synuclein PFF-induced ferroptosis in BV2 microglia. (**A**,**D**) Flow cytometric analysis of intracellular ROS levels in the control and siNrf2-transfected BV2 cells following PFF exposure (*n *= 3). (**B**,**E**) Flow cytometric assessment of lipid peroxidation in control and siNrf2-transfected BV2 cells after PFF treatment (*n *= 3). (**C**,**F**) Flow cytometric measurement of intracellular Fe^2+^ levels in the control and siNrf2-transfected BV2 cells following PFF exposure (*n *= 3). All experiments were independently repeated at least three times. Data were presented as means  ± SD. One-way analysis of variance (ANOVA) followed by Dunnett’s multiple comparison tests in (**D**–**F**). ** *p* < 0.01 and **** *p* < 0.0001.

To determine whether α-synuclein aggregation resulting from Nrf2 deficiency promotes ferroptosis, we treated the control and siNrf2 BV2 cells with PFFs and assessed the intracellular levels of ROS, lipid peroxidation, and Fe^2+^ by flow cytometry. PFF treatment alone significantly elevated these ferroptotic markers. Specifically, ROS levels increased from 7403 ± 28.62 to 16,017 ± 562.4 (*p* = 0.0001); lipid peroxidation increased from 15,230 ± 122.3 to 17,353 ± 109.1 (*p* = 0.0001); and the Fe^2+^ levels rose from 38,558 ± 1730 to 50,593 ± 146.3 (*p* = 0.0001). Notably, Nrf2 knockdown further exacerbated these effects, driving the ROS levels to 37,900 ± 586.5 (*p* = 0.0001), lipid peroxidation to 20,798 ± 223.2 (*p* = 0.0001), and Fe^2+^ levels to 55,672 ± 447.8 (*p* = 0.0017) (Figure 3A–F). These results demonstrate that Nrf2 deficiency intensifies PFF-induced oxidative stress, iron accumulation, and lipid peroxidation, thereby promoting ferroptosis-related phenotypes. Collectively, these data demonstrate that Nrf2 functions as a critical endogenous defense mechanism in BV2 microglia against α-synuclein PFF-induced ferroptosis-associated phenotypes. Loss of Nrf2 profoundly aggravates ferroptosis-like phenotypes by promoting pathological α-synuclein accumulation and aggregation, thereby amplifying oxidative stress, iron overload, and lipid peroxidation.

### 2.3. Activation of Nrf2 Attenuates α-Synuclein PFF-Induced Ferroptosis in BV2 Microglia

**Figure 4:**
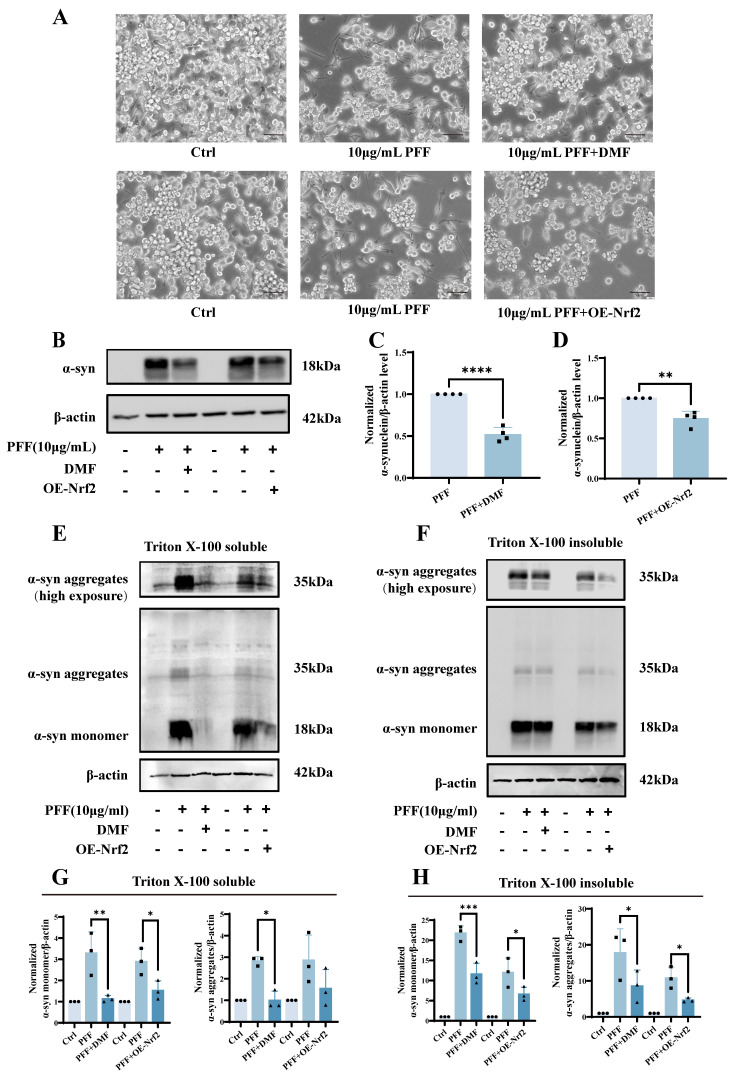
Activation of Nrf2 suppresses α-synuclein PFF-induced upregulation of intracellular α-synuclein expression. (**A**) Representative bright-field images of BV2 cells with lentiviral-mediated Nrf2 overexpression or DMF-mediated Nrf2 activation following treatment with 10 μg/mL PFF (scale bar: 50 μm). (**B**) Representative Western blot analysis showing changes in α-synuclein protein levels after total Nrf2 activation or overexpression. (*n *= 4). (**C**,**D**) Densitometric quantification of α-synuclein protein levels in total cell lysates. (**E**,**F**) Western blot analysis of α-synuclein in Triton X-100-soluble and Triton X-100-insoluble fractions following total Nrf2 activation or overexpression (*n *= 3). (**G**,**H**) Quantitative analysis of α-synuclein protein levels in the Triton X-100-soluble and Triton X-100-insoluble fractions. All experiments were independently repeated at least three times. Data were presented as means ± SD. Statistical significance was determined by the two-tailed Student’s *t*-test in (**C**,**D**). One-way analysis of variance (ANOVA) followed by Dunnett’s multiple comparison tests in (**H**). Kruskal–Wallis test with Dunn’s post hoc test in (**G**). * *p* < 0.05, ** *p* < 0.01, *** *p* < 0.001, and **** *p* < 0.0001.

Building on our observation that Nrf2 deficiency exacerbates ferroptosis, we next investigated whether activation of Nrf2 could protect BV2 microglial cells against α-synuclein PFF-induced injury. To determine the effect of Nrf2 overexpression on intracellular α-synuclein levels, BV2 cells were transduced with a lentiviral vector to overexpress the total Nrf2 and subsequently exposed to equal doses of α-synuclein PFF in both the control and Nrf2-overexpressing cells. Western blot analysis of the total cell lysates revealed that compared with the controls, Nrf2 overexpression significantly reduced the total α-synuclein levels to 0.7462 ± 0.09116 (*p* = 0.0014) (Figure 4A–D). Consistently, BV2 cells pretreated with 10 μM dimethyl fumarate (DMF) for 2 h prior to PFF exposure exhibited a marked reduction in total α-synuclein protein levels, which decreased to 0.5186 ± 0.08373 (*p* = 0.0001) relative to the PFF-treated group. These results indicate that enhancement of Nrf2 activity effectively reduces PFF-induced total α-synuclein levels.

To further assess the impact of Nrf2 activation on the aggregation state of α-synuclein, two complementary strategies—pharmacological activation with DMF and viral overexpression using AAV—were employed to induce the total Nrf2 expression in BV2 cells, followed by PFF treatment. Triton X-100-soluble and -insoluble fractions were subsequently extracted and analyzed by Western blotting. Both Nrf2 activation approaches markedly attenuated the PFF-induced accumulation of intracellular α-synuclein monomers and aggregates. Compared with PFF treatment alone, DMF-mediated Nrf2 activation reduced the soluble α-synuclein monomers from 3.292 ± 0.9994 to 1.138 ± 0.1594 (*p* = 0.0011) and soluble aggregates from 2.826 ± 0.2042 to 1.006 ± 0.4213 (*p* = 0.0404), insoluble monomers from 21.77 ± 1.872 to 11.62 ± 2.672 (*p* = 0.0003), and insoluble aggregates from 17.82 ± 6.642 to 8.589 ± 4.397 (*p* = 0.0272), respectively. Similarly, lentiviral-mediated Nrf2 overexpression diminished PFF-induced α-synuclein accumulation, reducing the soluble monomers from 2.896 ± 0.6208 to 1.533 ± 0.4486 (*p* = 0.0284), soluble aggregates from 2.863 ± 1.166 to 1.551 ± 0.8790 (*p* = 0.8045), insoluble monomers from 12.01 ± 3.587 to 6.606 ± 1.767 (*p* = 0.0326), and insoluble aggregates from 10.84 ± 3.007 to 4.679 ± 0.5905 (*p* = 0.0253) (Figure 4E–H).

**Figure 5:**
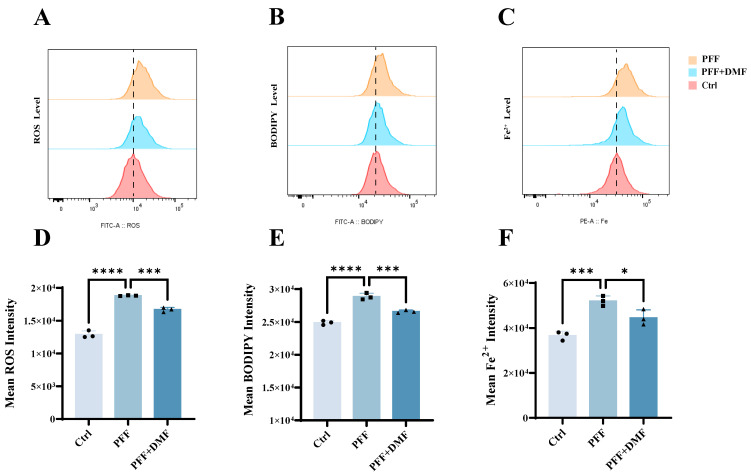
Activation or overexpression of Nrf2 attenuates α-synuclein PFF-induced ferroptosis in BV2 microglia. (**A**,**D**) Effects of DMF pretreatment on PFF-induced intracellular ROS levels in BV2 cells (*n *= 3). (**B**,**E**) Effects of DMF pretreatment on PFF-induced lipid peroxidation levels in BV2 cells (*n *= 3). (**C**,**F**) Effects of DMF pretreatment on PFF-induced intracellular Fe^2+^ accumulation in BV2 cells (*n *= 3). All experiments were independently repeated at least three times. Data were presented as means ± SD. One-way analysis of variance (ANOVA) followed by Dunnett’s multiple comparison tests in (**D**–**F**). * *p* < 0.05, *** *p* < 0.001, and **** *p* < 0.0001.

To investigate whether Nrf2 activation inhibits ferroptosis in BV2 cells induced by α-synuclein preformed fibrils (PFFs), flow cytometry was performed in the present study to measure the intracellular ROS levels, Fe^2+^ accumulation, and lipid peroxidation in the control group, the PFF-treated group, and the PFF + DMF group. The results showed that compared with the control group, PFF treatment alone significantly induced elevated ROS, increased lipid peroxidation, and Fe^2+^ overload. Specifically, ROS levels increased from 12,903 ± 560.9 to 18,811 ± 65.96 (*p* = 0.0001), lipid peroxidation increased from 24,897 ± 322.1 to 28,863 ± 501.1 (*p* = 0.0001), and Fe^2+^ levels increased from 36,648 ± 1967 to 52,002 ± 2248 (*p* = 0.0007). Compared with the PFF-treated group, Nrf2 activation effectively reversed these ferroptosis-related phenotypes: ROS levels were reduced to 16,686 ± 339.6 (*p* = 0.0009), lipid peroxidation was decreased to 26,592 ± 212.5 (*p* = 0.0005), and Fe^2+^ accumulation was lowered to 44,649 ± 3413 (*p* = 0.0244) (Figure 5A–F). Taken together, Nrf2 activation can effectively reverse the ferroptosis-associated characteristics (Fe^2+^ accumulation and elevated lipid peroxidation) induced by α-synuclein PFF in BV2 cells and downregulate cellular ROS levels, confirming that Nrf2 activation exerts a significant inhibitory effect on PFF-mediated ferroptosis-like changes in BV2 cells.

### 2.4. Overexpression of Nrf2 Mitigates α-Synuclein PFF-Induced Pathology and Behavioral Deficits in Parkinson’s Disease Model Mice

**Figure 6:**
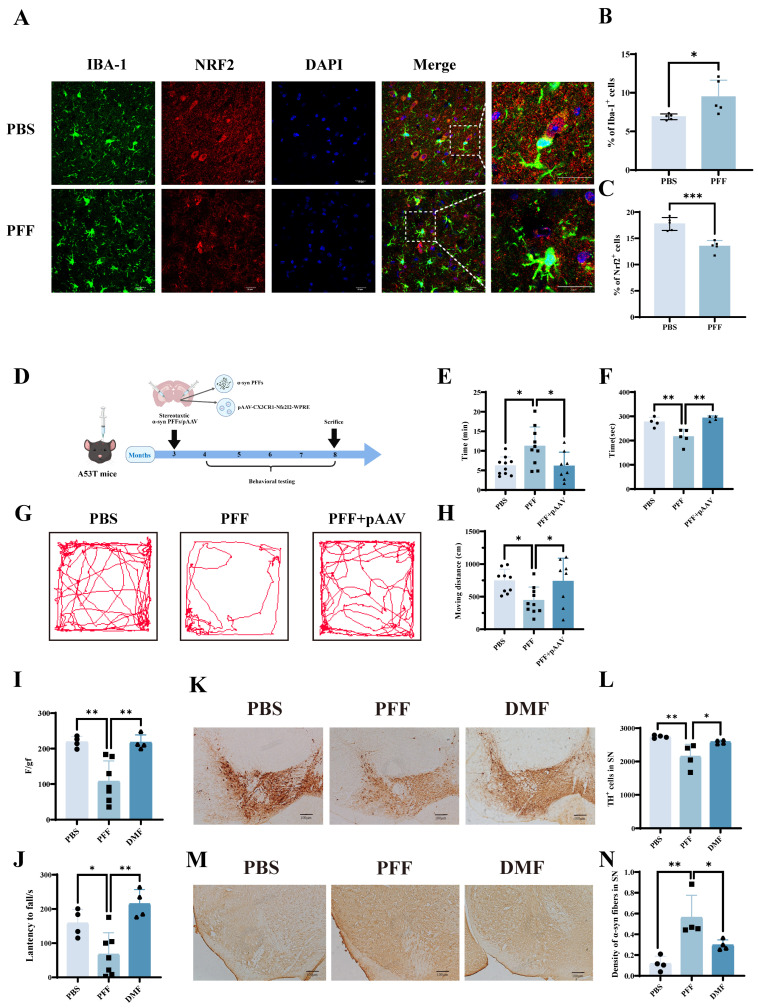
Activation or overexpression of Nrf2 mitigates α-synuclein PFF-induced pathology and behavioral deficits in Parkinson’s disease model mice. (**A**) Representative immunofluorescence images showing Iba-1 (microglia) and Nrf2 expression in the substantia nigra (SN) of PD model mice following α-synuclein PFF injection (scale bar: 20 μm; *n *= 5 mice per group). (**B**) Quantification of Iba-1-positive microglia in the substantia nigra of PBS- and PFF-treated mice. (**C**) Quantitative analysis of Nrf2-positive cells in the substantia nigra of the PBS-and PFF-treated mice. (**D**) Schematic overview of the experimental design. (**E**) Olfactory performance in PD mice with microglia-specific Nrf2 overexpression (PBS group, *n *= 10; PFF group, *n *= 10; PFF + pAAV group, *n *= 8). (**F**) Rotarod performance evaluating motor coordination in PD mice with microglia-specific Nrf2 overexpression (PBS group, PFF group, PFF + pAAV group: *n *= 4, 5, 4 per group, respectively). Movement trajectories (**G**) and moving distances (**H**) were recorded during the open-field test assessing spontaneous locomotor activity in PD mice with microglia-specific Nrf2 overexpression (PBS group, PFF group, PFF + pAAV group: *n *= 9, 10, 8 per group, respectively). (**I**) Effects of DMF-mediated Nrf2 activation on forelimb grip strength in PD mice. (**J**) Effects of DMF-mediated Nrf2 activation on rotarod performance in PD mice (PBS group, *n *= 4; PFF group, *n *= 7; DMF group, *n *= 4). (**K**,**L**) Tyrosine hydroxylase (TH) immunohistochemical staining showing dopaminergic neuron survival in the substantia nigra, including representative images (**K**) and quantitative analysis (**L**) (scale bar, 100 μm; *n *= 4). (**M**,**N**) α-Synuclein immunohistochemical staining of the striatum illustrating α-synuclein aggregation, including representative images (**M**) and mean optical density quantification (**N**) (scale bar, 100 μm; *n *= 4). Data were presented as means ± SD. One-way analysis of variance (ANOVA) followed by Dunnett’s multiple comparison tests in (**E**,**F**,**H**–**J**,**L**,**N**), asterisks (*) indicate statistically significant differences relative to the PFF control group (PBS vs. PFF or PFF vs. DMF).* *p* < 0.05, ** *p* < 0.01, and *** *p* < 0.001.

Collectively, these findings establish total Nrf2 as a critical endogenous defense factor in BV2 microglia against α-synuclein PFF-induced ferroptosis. To determine whether microglial Nrf2 expression is altered under Parkinson’s disease (PD) pathological conditions in vivo, α-synuclein PFF were bilaterally injected into the brains of A53T transgenic mice. Following disease onset, brain tissues were harvested, cryosectioned, and subjected to immunofluorescence co-staining. Compared with the control mice, PD model mice exhibited a significant increase in the proportion of Iba-1-positive microglia in the substantia nigra (SN), which rose from 6.887 ± 0.3734 to 9.459 ± 2.177 (*p* = 0.0314). These microglia displayed an activated morphology characterized by enlarged cell bodies and shortened processes (Figure 6A,B). In parallel, Nrf2 expression in the SN was markedly reduced, decreasing from 17.74 ± 1.234 to 13.49 ± 1.088 (*p* = 0.0004) (Figure 6C). These observations indicate that α-synuclein PFF-induced PD pathology is associated with microglial activation and concomitant downregulation of Nrf2, consistent with in vitro findings.

To further investigate whether the upregulation of microglial Nrf2 confers neuroprotection in PD mice, an AAV vector encoding Nrf2 was constructed and stereotaxically co-injected into the bilateral SN of A53T transgenic mice at the time of PFF administration (Figure 6D). To confirm that the pAAV–CX3CR1–Nrf2 vector achieved microglia-restricted Nrf2 upregulation, we performed immunofluorescence co-staining for Nrf2 and cell-type-specific markers in the substantia nigra of mice that received AAV injection. As shown in Supplementary Figure S1, strong Nrf2 immunoreactivity was detected in Iba-1-positive microglia. These results demonstrate that the CX3CR1 promoter driven AAV mediates efficient and selective Nrf2 overexpression in microglia. Long-term behavioral analyses were then performed to evaluate the in vivo effects of Nrf2 pathway activation. Behavioral testing revealed that PFF-induced PD mice displayed a significant impairment in olfactory function, with latency values increased from 6.107 ± 2.332 to 11.13 ± 4.965 (*p* = 0.0128), whereas CX3CR1-promoter-driven Nrf2 overexpression significantly restored olfactory performance 6.050 ± 3.597 (*p* = 0.0176) (Figure 6E). In the open-field test, PFF-treated mice exhibited a marked reduction in spontaneous locomotor activity, which declined from 737.7 ± 182.6 to 439.1 ± 207.8 (*p* = 0.0331); this deficit was significantly reversed by microglial Nrf2 overexpression, with activity levels increasing to 730.3 ± 360.1 (*p* = 0.0449) (Figure 6G,H). Given the presence of prominent non-motor deficits, including olfactory dysfunction and reduced spontaneous activity, in PFF-induced PD mice, we next assessed motor coordination using the rotarod test. PFF administration significantly shortened the latency from 275.6 ± 20.22 to 214.5 ± 32.07 (*p* = 0.0063), indicating pronounced motor impairment. Notably, AAV-mediated Nrf2 overexpression not only ameliorated non-motor deficits but also significantly improved motor performance, extending the rotarod latency to 291.4 ± 10.50 (*p* = 0.0014) (Figure 6F). These results collectively demonstrate that increasing microglial Nrf2 expression is associated with protective effects against both motor and non-motor dysfunctions in PD model mice.

To further evaluate the in vivo effects of the Nrf2 activator dimethyl fumarate (DMF), A53T transgenic mice were stereotaxically injected with α-synuclein PFF to establish a PD model, followed by daily oral administration of DMF (30 mg/kg) for one month. Behavioral and histological assessments were subsequently performed. In the grip-strength test, PFF-treated PD mice exhibited a marked reduction in forelimb grip strength, which declined from 218.6 ± 16.16 to 107.3 ± 58.05 (*p* = 0.0038). DMF treatment significantly reversed this deficit, restoring grip strength to 217.4 ± 21.09 (*p *= 0.0041) (Figure 6I). Similarly, rotarod testing revealed that PFF exposure profoundly impaired motor coordination, with latency to fall reduced from 158.3 ± 40.37 to 67.07 ± 63.27 (*p* = 0.0448). DMF administration markedly improved motor performance, increasing rotarod latency to 215.0 ± 41.61 (*p* = 0.0022) (Figure 6J). Histopathological analyses further supported the behavioral findings. Tyrosine hydroxylase (TH) immunohistochemistry demonstrated a significant loss of dopaminergic neurons in the substantia nigra of PD mice compared with the controls, with neuronal counts reduced from 2744 ± 42.05 to 2149 ± 368.4 (*p* = 0.0068). DMF treatment significantly attenuated dopaminergic neuron loss, increasing TH-positive neuron numbers to 2585 ± 55.83 (*p* = 0.0342) (Figure 6K,L). In parallel, α-synuclein immunohistochemical staining in the striatum revealed a pronounced accumulation of α-synuclein-positive aggregates in PD model mice, rising from 0.1173 ± 0.07048 to 0.5618 ± 0.2144 (*p* = 0.0020). Notably, DMF treatment markedly reduced α-synuclein immunoreactivity and aggregate burden to 0.2967 ± 0.05000 (*p* = 0.0366) (Figure 6M,N).

Taken together, these data demonstrate that both microglia-targeted Nrf2 overexpression and systemic treatment with the Nrf2 activator DMF robustly ameliorate α-synuclein PFF-induced motor and non-motor deficits, attenuate dopaminergic neurodegeneration, and reduce pathological α-synuclein aggregation in PD model mice. These findings suggest that Nrf2 activation represents a promising therapeutic strategy for modifying PD progression, with its protective effects likely involving, at least in part, the modulation of microglial function.

## 3. Discussion

Parkinson’s disease (PD) is one of the most prevalent neurodegenerative disorders, affecting approximately 1–4% of the population over 65 years of age [21]. The pathological hallmarks of PD are the progressive loss of dopaminergic neurons in the substantia nigra pars compacta (SNpc) and the abnormal aggregation of α-synuclein into Lewy bodies. Despite the well-established nature of these features, the precise molecular mechanisms driving neuronal degeneration remain incompletely understood. In recent years, ferroptosis, an iron-dependent form of regulated cell death driven by lipid peroxidation, has garnered increasing attention for its role in the pathogenesis of PD [22].

Emerging evidence suggests that microglia are more susceptible to ferroptosis than neurons or astrocytes, a vulnerability that may impair their capacity to phagocytose and clear α-synuclein [23]. Intranasal administration of PFF has been shown to induce iron deposition specifically in microglia within the substantia nigra, rather than in dopaminergic neurons or other glial cell types [24]. Furthermore, microglial exposure to α-synuclein triggers the release of interleukin-6 (IL-6), which in turn promotes neuronal iron uptake and subsequent cell death [25]. Ryan and colleagues demonstrated that microglia are critical drivers of ferroptosis-dependent neurodegeneration [26] In astrocyte–neuron co-cultures, significant neuronal lipid peroxidation was observed only in the presence of microglia; in microglia-free cultures, the ferroptotic process in neurons was markedly delayed. These findings collectively support the notion that microglial ferroptosis may serve as an early cellular event that initiates neuroinflammation within the dopaminergic system. In alignment with this hypothesis, our study demonstrates that α-synuclein PFF exposure reduces cell viability, downregulates Nrf2 expression, and elevates levels of ROS, Fe^2+^, and lipid peroxidation in BV2 microglial cells.

Microglia are resident phagocytes in the central nervous system, and they can help clear misfolded α-synuclein aggregates in Parkinson’s disease [27]. Previous studies have observed that microglia are involved in the propagation of pathological α-synuclein [28,29]. Microglia can phagocytose α-synuclein fibrils and process them into α-synuclein subtypes with higher seeding activity [30]. Therefore, studying the processing effect of microglia on α-synuclein PFFs helps to elucidate the pathogenesis of Parkinson’s disease. Through bidirectional verification via gene knockdown and overexpression, this study confirmed for the first time in microglia that the expression level of Nrf2 is negatively correlated with the aggregation degree of α-synuclein. Nrf2 deficiency promotes α-synuclein aggregation, whereas Nrf2 activation inhibits it. This finding expands the traditional antioxidant function of Nrf2, suggesting that Nrf2 may be a key regulator in maintaining α-synuclein protein homeostasis, and provides a new molecular node for understanding the vicious cycle between oxidative stress and α-synuclein pathology in PD.

Nuclear factor erythroid 2-related factor 2 (Nrf2) is a master transcriptional regulator of the cellular antioxidant defense system. Nuclear factor erythroid 2 related factor 2 (Nrf2) plays a critical role in inhibiting ferroptosis by regulating the expression of downstream target genes, including glutathione peroxidase 4 (GPX4), heme oxygenase 1 (HO-1), and ferritin heavy chain 1 (FTH1) [31]. Previous studies have established that Nrf2 modulates microglial activation and mitigates neuroinflammation in the MPTP mouse model of PD [32,33,34]. It is also well-documented that Nrf2 levels decline with age, and an irreversible reduction in Nrf2 protein expression has been observed in both aging and neurodegenerative conditions such as Alzheimer’s disease [35,36]. The expression dynamics of Nrf2 in the brains of patients with Parkinson disease remain controversial. Two opposing hypotheses have been proposed, namely compensatory upregulation and terminal exhaustion. On the one hand, studies have reported pronounced nuclear translocation of Nrf2 in surviving nigral neurons of PD patients, suggesting its activation [37]. Similarly, short-term (2-h) exposure to α-synuclein in BV2 cells has been shown to transiently elevate Nrf2 protein levels [38], likely as an adaptive response to drive the expression of antioxidant enzymes such as HO-1, thereby counteracting oxidative stress and reducing ROS. On the other hand, as pathology progresses, Nrf2 levels appear to decline sharply. Our findings are consistent with this latter phase: following 72 h of α-synuclein PFF exposure, BV2 cells exhibited a marked downregulation of Nrf2, coinciding with the onset of ferroptosis-associated phenotypes. This suggests that sustained α-synuclein PFF stress ultimately depletes Nrf2, compromising the antioxidant capacity of microglia and rendering them vulnerable to ferroptosis-associated phenotypes.

Despite the ongoing debate regarding its temporal expression patterns, there is consensus that activating the Nrf2 pathway confers neuroprotection in PD, whereas its deficiency accelerates disease progression. For example, transgenic activation of Nrf2 through Nrf2 overexpression combined with Keap1 silencing has been demonstrated to alleviate dopaminergic neuron loss and motor dysfunction in an α-synuclein-induced Drosophila model of Parkinson’s disease [39]. Conversely, Nrf2 knockout exacerbates dopaminergic neurodegeneration and motor dysfunction in α-synuclein-overexpressing mice. Moreover, stereotaxic delivery of adeno-associated virus (rAAV6-α-SYN) encoding human α-synuclein into the substantia nigra of Nrf2-deficient mice results in more severe dopaminergic neuron degeneration and heightened neuroinflammation compared to wild-type controls [38]. These studies underscore the essential neuroprotective function of Nrf2. Our results not only corroborate these findings but also extend them by demonstrating that Nrf2 knockdown exacerbates α-synuclein aggregation and ferroptosis-associated phenotypes, while Nrf2 overexpression exerts protective effects. Importantly, we identify microglia as key cellular mediators through which Nrf2 regulates α-synuclein pathology, likely by modulating the ferroptotic cascade. Our findings establish a novel functional link between Nrf2 and α-synuclein homeostasis, suggesting that Nrf2 plays an integral role in maintaining α-synuclein proteostasis.

In vivo, microglia-specific Nrf2 overexpression or systemic DMF administration significantly improved motor impairments, olfactory deficits, and reduced spontaneous locomotor activity in Parkinson’s disease model mice, preserved nigral dopaminergic neurons, and diminished α-synuclein aggregation in the striatum. These interventions significantly preserved nigral dopaminergic neurons and mitigated α-synuclein pathological accumulation. As a clinically approved drug, DMF has garnered increasing attention for its neuroprotective potential in neurodegenerative disorders. In SH-SY5Y cells, DMF has been shown to reduce intracellular ROS levels and protect against 6-hydroxydopamine (6-OHDA)-induced cytotoxicity [40]. Moreover, DMF treatment improves mitochondrial and synaptic integrity in primary hippocampal neurons derived from A53T transgenic mice [41]. In PD models driven by AAV-mediated α-synuclein overexpression, DMF administration alleviates motor impairments and reduces dopaminergic neuron loss [42]. Consistent with these prior studies, we employed an α-synuclein PFF-injected A53T transgenic mouse model and demonstrated that DMF treatment markedly improves motor performance and preserves nigral dopaminergic neurons. Importantly, our study extends these therapeutic observations to microglia, revealing that microglia-targeted Nrf2 overexpression mediated by pAAV-CX3CR1-Nfe2l2-WPRE is sufficient to confer pronounced neuroprotection. This provides direct experimental evidence supporting a central role for microglia–neuron interactions in PD pathogenesis. In line with our findings, Heurtaux et al. reported that apomorphine attenuates A53T α-synuclein-induced microglial reactivity by activating the Nrf2 signaling pathway, thereby reducing proinflammatory polarization and restoring phagocytic function [43]. Together, these converging lines of evidence further underscore the therapeutic promise of targeting microglial Nrf2 signaling in PD. We acknowledge that while DMF is widely used as a pharmacological activator of Nrf2, it may also exert Nrf2-independent effects. Moreover, systemic administration of DMF in vivo can potentially act on multiple cell types beyond microglia, including neurons, astrocytes, endothelial cells, and peripheral immune cells. Therefore, the neuroprotective effects observed in DMF-treated PD mice cannot be solely attributed to Nrf2 activation in microglia. It is likely that DMF confers broader anti-inflammatory, antioxidant, and immunomodulatory actions that collectively contribute to the preservation of dopaminergic neurons and the improvement of behavioral outcomes. Our genetic loss-of-function (Nrf2 knockdown in vitro) and gain-of-function (microglia-specific Nrf2 overexpression in vivo) experiments support a role for microglial Nrf2 in protection; however, the contribution of Nrf2-independent pathways and actions on other cell types remains to be dissected in future studies using cell-type-specific Nrf2 knockout mice or more selective Nrf2 activators.

However, we frankly acknowledge that the specific molecular mechanism by which Nrf2 regulates α-synuclein aggregation has not yet been clarified, which represents one of the main limitations of this study. Studies have shown that intracellular α-synuclein is primarily degraded through the coordinated action of the autophagy–lysosome pathway (ALP) and the ubiquitin–proteasome system (UPS) [44]. Meanwhile, it has been reported that Nrf2 can regulate redox balance by clearing ROS and modulate proteasome function and autophagy pathways, enabling it to sense and respond to the accumulation of misfolded or aggregated proteins within cells [45]. Therefore, subsequent research will systematically investigate whether Nrf2 is involved in regulating the clearance of α-synuclein through pathways such as autophagy. Furthermore, Nrf2 activation may alleviate lipid peroxidation and iron accumulation by upregulating GPX4 and FTH1 [46]. This may further alleviate α-synuclein pathology. Conversely, based on the existing literature [47], we hypothesize that α-synuclein PFFs may induce the degradation of Nrf2 by promoting the binding of Keap1 to Nrf2 or interfering with the p62–Keap1–Nrf2 axis. Future studies are required to verify these hypotheses experimentally, including cycloheximide chase assays and target gene interference. We acknowledge that we only assessed the total Nrf2 protein levels in whole-cell lysates and did not perform cytoplasmic-nuclear fractionation. Therefore, we cannot definitively conclude that the observed changes reflect Nrf2 nuclear translocation or transcriptional activation. This study has the limitation of not providing experimental data on the direct rescue effect of ferroptosis inhibitors, and it is clarified that the research conclusions are drawn solely based on phenotypic characteristics and biochemical detection evidence. It should be noted that we did not directly evaluate the expression of canonical Nrf2 target genes (e.g., HO-1, NQO1) in microglia under PD-relevant conditions. Therefore, the transcriptional activation of Nrf2 in our model remains to be formally validated, and this constitutes a limitation of the present study.

In summary, our study delineates a previously unrecognized mechanism by which Nrf2 activation counteracts α-synuclein PFF-induced neurotoxicity through the inhibition of microglial ferroptosis-associated phenotypes. These findings identify Nrf2 as a compelling therapeutic target and propose a rational strategy for PD intervention. Future investigations should expand upon these results by exploring deeper mechanistic pathways, validating efficacy across diverse disease models, and rigorously assessing translational feasibility, thereby facilitating the clinical advancement of Nrf2-targeted therapies for Parkinson’s disease.

## 4. Materials and Methods

### 4.1. Cell Culture and Treatment

The murine microglial BV2 cell line was obtained from the Shanghai Institute of Materia Medica. Cells were maintained in high-glucose DMEM (Basalmedia, L110KJ, Shanghai, China) supplemented with 10% fetal bovine serum (FBS, Gibco, A5669701, Auckland, New Zealand) and 1% penicillin–streptomycin (Basalmedia, S110JV, Shanghai, China). Cultures were incubated at 37 °C in a humidified atmosphere containing 5% CO_2_. Cells were passaged by gentle trituration when confluence reached approximately 80–90%.

### 4.2. Preparation of α-Synuclein Preformed Fibrils (PFFs)

PFFs were prepared according to the experimental method reported previously [48]. Recombinant human α-synuclein monomer (CF66, Novoprotein, Shanghai, China) was diluted to 5 mg/mL in sterile PBS and incubated at 37 °C with constant shaking at 1000 rpm for 7 days. The resulting PFFs were aliquoted and stored at −80 °C until use. Immediately prior to the experiments, PFFs were thawed at room temperature, diluted to the working concentration with sterile PBS, and sonicated using a probe sonicator at 20% amplitude for 30 s (0.5 s on/0.5 s off pulses) to generate uniform short fibril seeds suitable for cellular or in vivo administration. For each batch of PFFs used, we verified the formation of β-sheet structures by the ThT fluorescence method. All experiments were conducted using the same batch of prefabricated protofibers to avoid batch-to-batch variations.

### 4.3. DMF Treatment on BV2 Cells

BV2 cells were seeded in 6-well plates at a density of 8 × 10^4^ cells/mL. After 24 h of adherent culture, cells were pre-incubated with DMF at a final concentration of 10 μM for 3 h. Thereafter, the medium was replaced with fresh complete medium (without DMF), and then the cells were challenged with α-synuclein PFFs (10 μg/mL) for an additional 72 h. Thus, DMF was present only during the 3 h pre-treatment period and was not maintained during the subsequent PFF exposure.

### 4.4. Nrf2 Knockdown and Overexpression in BV2 Cells

For Nrf2 knockdown, small interfering RNA (siRNA) targeting mouse Nrf2 and negative control siRNA were designed and synthesized by Tsingke Biotechnology (Beijing, China). BV2 cells seeded in 6-well plates at 30% confluence were transfected with siRNA using Lipofectamine™ RNAiMAX Transfection Reagent (Thermo Fisher Scientific, 13778100, Carlsbad, CA, USA). The Nrf2 siRNA sequences (5′→3′) were: sense, GCAAGAAGCCAGAUACAAAGA; antisense, UUUGUAUCUGGCUUCUUGCUU. Culture medium was replaced with fresh DMEM 10 h post-transfection.

For Nrf2 overexpression, BV2 cells at 30% confluence in 6-well plates were transduced with the pSlenti-EF1-EGFP-P2A-Puro-CMV-MCS-3XFLAG-Nfe2L2-EPRE lentiviral vector (H10420, OBiO Technology, Shanghai, China). Medium was refreshed 24 h after transduction.

### 4.5. Cell Viability Assay

BV2 cells in logarithmic growth phase were seeded into 96-well plates at a density of 5 × 10^3^ cells per well. After 24 h of culture to allow attachment, the medium was replaced with fresh medium containing varying concentrations of α-synuclein PFFs (0, 1, 3, 5, or 10 μg/mL) for 72 h. Subsequently, 90 μL of fresh medium and 10 μL of Cell Counting Kit-8 (CCK-8) reagent (AC11L054, HZYMES, Wuhan, China) were added to each well, and plates were incubated for an additional 2 h. Absorbance at 450 nm was measured using a microplate reader (SpectraMax Plus 384, Molecular Devices, San Jose, CA, USA). Cell viability (%) was calculated as (OD value of treatment group/OD value of control group) × 100%.

### 4.6. Western Blotting

Cells were lysed in RIPA buffer (Epizyme Biotech, Cambridge, MA, USA) supplemented with protease (GFR101, Epizyme Biotech, Shanghai, China) and phosphatase (GFR102, Epizyme Biotech, Shanghai, China) inhibitors on ice for 30 min, followed by centrifugation at 15,000 rpm for 15 min at 4 °C. Supernatants were collected as total protein extracts. For Triton X-100-soluble and -insoluble α-synuclein fractionation, cells were lysed in TBS containing 2% Triton X-100 and inhibitors, followed by centrifugation at 15,000 rpm for 15 min at 4 °C. The supernatant was collected as the soluble fraction. Pellets were resuspended in TBS containing 1% SDS and inhibitors to obtain the insoluble fraction. Protein samples were denatured at 100 °C for 10 min, separated on 12.5% SDS–PAGE gels (PG113, Epizyme Biotech, Shanghai, China), and transferred onto nitrocellulose membranes using a wet transfer system. Membranes were blocked with 5% non-fat milk for 2 h at room temperature and incubated with primary antibodies overnight at 4 °C. After incubation with HRP-conjugated secondary antibodies for 2 h at room temperature, immunoreactive bands were visualized using enhanced chemiluminescence (ECL, 99883–11, YLESA, Shanghai, China). Band intensities were quantified using ImageJ (Version 1.54f, National Institutes of Health, Bethesda, MD, USA) and normalized to β-actin.

Primary antibodies included anti-NRF2 (1:1000, D1Z9C, Cell Signaling Technology, Danvers, MA, USA), anti-β-actin (1:5000, S0B0005, Starter, Hangzhou, China), and purified mouse anti-α-synuclein (1:1000, 610786, BD Transduction Laboratories, San Jose, CA, USA).

### 4.7. Measurement of Intracellular ROS Levels

Total intracellular ROS levels were assessed using the fluorescent probe H2DCFDA (D399, Thermo Fisher Scientific, Waltham, MA, USA). Treated BV2 cells were washed once with PBS and incubated with 1 μM H2DCFDA at 37 °C for 30 min in the dark, with gentle mixing every 5 min. Cells were washed three times with PBS, resuspended in 500 μL PBS, and immediately analyzed using a Cytek^®^ Aurora full-spectral flow cytometer (Cytek Biosciences, Fremont, CA, USA). Data were processed using FlowJo software (Version 10.8.1, BD Life Sciences, Ashland, OR, USA).

### 4.8. Measurement of Intracellular Ferrous Iron Levels

Intracellular ferrous iron (Fe^2+^) levels were detected using FerroOrange (F374, Dojindo, Kumamoto, Japan). Cells were washed once with PBS and incubated with 0.1 μM FerroOrange at 37 °C for 30 min in the dark, followed by immediate flow cytometric analysis on a Cytek^®^ Aurora full-spectral flow cytometer (Cytek Biosciences, Fremont, CA, USA). Data were analyzed using FlowJo.

### 4.9. Measurement of Lipid Peroxidation

Lipid peroxidation was assessed using the BODIPY™ 581/591 C11 probe (D3861, Thermo Fisher Scientific). Treated BV2 cells were collected and incubated with 4 μM BODIPY™ 581/591 C11 working solution at 37 °C for 30 min in the dark. After incubation, cells were washed three times with PBS, resuspended in PBS, and immediately analyzed by Cytek^®^ Aurora full-spectral flow cytometer (Cytek Biosciences, Fremont, CA, USA). Data were processed using FlowJo software.

### 4.10. Animals and Housing

A53T α-synuclein transgenic mice (M83 line; Tg(SNCA)83Vle) were purchased from the Shanghai Model Organisms Center. Mice were housed under specific pathogen-free conditions at 24 ± 2 °C with a 12 h light/dark cycle (lights on from 09:00 to 21:00) and 50–60% relative humidity. Food and water were provided ad libitum, with no more than six mice per cage. All animal studies were performed according to protocols that were reviewed and approved by the Institutional Animal Care and Use Committee at the Shanghai Institute of Materia Medica (IACUC No: 2022-10-GMY-30).

### 4.11. AAV Vector and Microglia-Specific Nrf2 Overexpression

To achieve microglia-restricted overexpression of Nrf2, a recombinant adeno-associated virus (AAV) vector serotype 2/9 (AAV2/9) was constructed, incorporating the mouse CX3CR1 promoter (pAAV-CX3CR1-Nfe2l2-WPRE) to drive microglia-specific expression of mouse Nrf2 (Nfe2l2). The virus was packaged and titrated by Heyuan Biotech (Songyuan, China). The final titer was determined by qPCR as 2.69 × 10^12^ viral genomes (vg)/mL. For stereotaxic injection, 2 μL of the AAV solution (diluted to 1 × 10^12^ vg/mL in sterile PBS) was bilaterally injected into the substantia nigra.

### 4.12. Stereotaxic Injection

Three-month-old A53T transgenic female mice were randomly assigned to three groups: PBS, PFF, and PFF + pAAV-CX3CR1-Nfe2l2-WPRE. Following anesthesia, mice were secured in a stereotaxic frame (68807, RWD Life Science, Shenzhen, China), and the surgical site was shaved and disinfected. Using a microsyringe, 1 μL of α-synuclein PFFs (5 μg/μL) was bilaterally injected into the substantia nigra pars compacta at a rate of 0.2 μL/min. The needle was retained in place for 5 min before withdrawal to allow diffusion. Control mice received PBS injections at identical coordinates: AP −3.10 mm, ML ±1.30 mm, DV −4.75 mm.

For microglia-specific Nrf2 overexpression, pAAV-CX3CR1-Nfe2l2-WPRE was injected at 0.5 μL per site using the same stereotaxic coordinates and injection rate. The PBS and PFF groups received equal volumes of PBS. Mice were allowed to recover and maintained under standard housing conditions. A total of 28 mice were used (PBS: *n* = 10, PFF: *n* = 10, PFF + pAAV-CX3CR1-Nfe2l2-WPRE: *n* = 8).

### 4.13. DMF Administration

A53T mice were divided into PBS, PFF, and PFF + DMF groups. Stereotaxic injections were performed as described above. Dimethyl fumarate (DMF; MCE, HY-Y0345) was dissolved in 0.5% carboxymethylcellulose sodium (CMC-Na). Beginning two days after PFF injection, mice received daily oral gavage of DMF at a dose of 30 mg/kg for three consecutive months. Control groups received equivalent volumes of vehicle.

### 4.14. Behavioral Assessments

Rotarod Test: Motor coordination was evaluated using an accelerating rotarod apparatus. Mice were placed on the rotating rod, which accelerated from 5 to 40 rpm over 5 min. The latency to fall was recorded. Each mouse underwent three trials with 30 min inter-trial intervals, and the mean value was calculated.

Open Field Test: Mice were placed in the center of a 40 cm × 40 cm × 40 cm arena divided into 4 equal squares. Locomotor activity was recorded for 7 min using a video-tracking system, and the total distance traveled during the final 5 min was analyzed by EthoVision^®^ XT (Noldus, Wageningen, The Netherlands).

Grip Strength Test: Forelimb grip strength was measured using a grip strength meter. Mice were allowed to grasp the grid with their forepaws and were gently pulled backward by the tail until release. The maximal force was recorded. Three measurements were averaged and normalized to body weight.

Buried Food Test: Olfactory function was assessed following 12 h of food deprivation. A single food pellet was buried 1 cm beneath the bedding at one of three predetermined locations. The latency to locate the pellet was recorded. Each mouse underwent three trials with 1 h intervals, and the average latency was calculated.

Behavioral tests were conducted in a single-blind manner, where the operators were unaware of the group allocation.

### 4.15. Immunofluorescence and Immunohistochemistry

Following behavioral testing, mice were perfused transcardially with 0.9% saline and fixed in 4% paraformaldehyde for 24 h. Brains were cryoprotected in 30% sucrose at 4 °C until sinking, embedded in OCT compound, and coronally sectioned at 20 μm using a cryostat (Leica, CM1950, Nussloch, Germany). Sections were stored at −20 °C in cryoprotectant solution.

For immunofluorescence, sections were washed in PBS, permeabilized with 0.3% Triton X-100 for 15 min, and blocked with 10% normal goat serum for 1 h at room temperature. Sections were incubated with primary antibodies overnight at 4 °C, followed by fluorophore-conjugated secondary antibodies for 1 h at room temperature in the dark. Sections were mounted with antifade medium containing DAPI and imaged using a confocal microscope (Olympus; FV3000, Tokyo, Japan). Photomicrographs were captured and analyzed with FV31S software. The primary antibodies used were: anti-Iba1 (1:1000, 019-19741, Wako, Osaka, Japan), Anti-Nrf2 antibody (1:200, PT0855R, Immunoway, Plano, TX, USA) The secondary antibodies used were: Alexa Fluor™ 555 (1:800, A-31570, Thermo Fisher Scientific, MA, USA) and Alexa Fluor™ 488 (1:800, A-21206, Thermo Fisher Scientific, MA, USA).

For immunohistochemistry, endogenous peroxidase activity was quenched with 3% H_2_O_2_ for 10 min. After blocking, sections were incubated with the primary antibodies anti-tyrosine hydroxylase (1:500, ab137869, Abcam, Cambridge, UK) or anti-α-synuclein (1:500, ab138501, Abcam) overnight at 4 °C, followed by incubation with SignalStain Boost IHC Detection Reagent (8125S, Cell Signaling Technology, MA, USA) at 37 °C for 1 h. Signals were developed using DAB, counterstained, and mounted with neutral resin. Images were acquired using a multispectral pathological imaging system (Vectra Polaris, Akoya, MA, USA) and analyzed with ImageJ for positive cell counts or mean optical density.

### 4.16. Statistical Analysis

All data presented in this study were obtained from at least three independent experiments and are expressed as mean ± standard deviation (SD). In the in vitro experiments, n represents the number of independent biological replicates. In the in vivo experiments, n indicates the number of mice per group, as specified in each figure legend. Technical replicates were averaged before statistical analysis. Statistical analyses and data visualization were performed using GraphPad Prism software (version 10.1). Comparisons between two groups were made using the two-tailed Student’s *t*-test. Multiple comparisons among groups were performed using one-way ANOVA followed by Dunnett’s test. For datasets where normality or variance homogeneity was violated, the non-parametric Kruskal–Wallis test with Dunn’s post hoc correction was used. For all statistical tests, statistical significance was set at * *p* < 0.05, ** *p* < 0.01, and *** *p* < 0.001.

## 5. Conclusions

This study demonstrates that PFFs induce ferroptosis-associated phenotypes in microglia by downregulating Nrf2 expression, whereas the activation of Nrf2 effectively reverses this pathological process. In vivo, both microglia-specific Nrf2 overexpression and systemic administration of the Nrf2 activator dimethyl fumarate (DMF) markedly ameliorated motor and non-motor deficits in Parkinson’s disease (PD) model mice, preserved nigral dopaminergic neurons, and attenuated pathological α-synuclein aggregation. Collectively, our findings uncover a previously unrecognized role for microglial ferroptosis in PD pathogenesis, identify Nrf2 as a central molecular regulator linking α-synuclein aggregation to ferroptotic cell death, and provide experimental support for the further evaluation of DMF as a potential disease-modifying therapeutic strategy for PD.

## Data Availability

Data will be made available on request.

## Supplementary Materials

The following supporting information can be downloaded at: https://www.mdpi.com/article/10.3390/ijms27104579/s1.

## Abbreviations

The following abbreviations are used in this manuscript:

PD	Parkinson’s disease
Nrf2	Nuclear factor erythroid 2-related factor 2
PFFs	Pre-formed fibrils
ROS	Reactive oxygen species
DMF	Dimethyl fumarate
DA	Dopaminergic
LBs	Lewy bodies
GPX4	Glutathione peroxidase 4
Keap1	Kelch-like ECH-associated protein 1
AREs	Antioxidant response elements
RRMS	Relapsing-remitting multiple sclerosis
AD	Alzheimer’s disease
SNpc	Substantia nigra pars compacta
IL-6	Interleukin-6
FTH1	Ferritin heavy chain 1
HO-1	Heme oxygenase 1
6-OHDA	6-hydroxydopamine
ALP	Autophagy–lysosome pathway
UPS	Ubiquitin–proteasome system
