# Supplementary figures and images for: Microglial Nrf2 Activation Orchestrates Ferroptosis Inhibition and α-Synuclein Clearance in Parkinson’s Disease
Source: Int J Mol Sci. 2026 May 20;27(10):4579. doi: 10.3390/ijms27104579 (PMC13207490; doi:10.3390/ijms27104579)

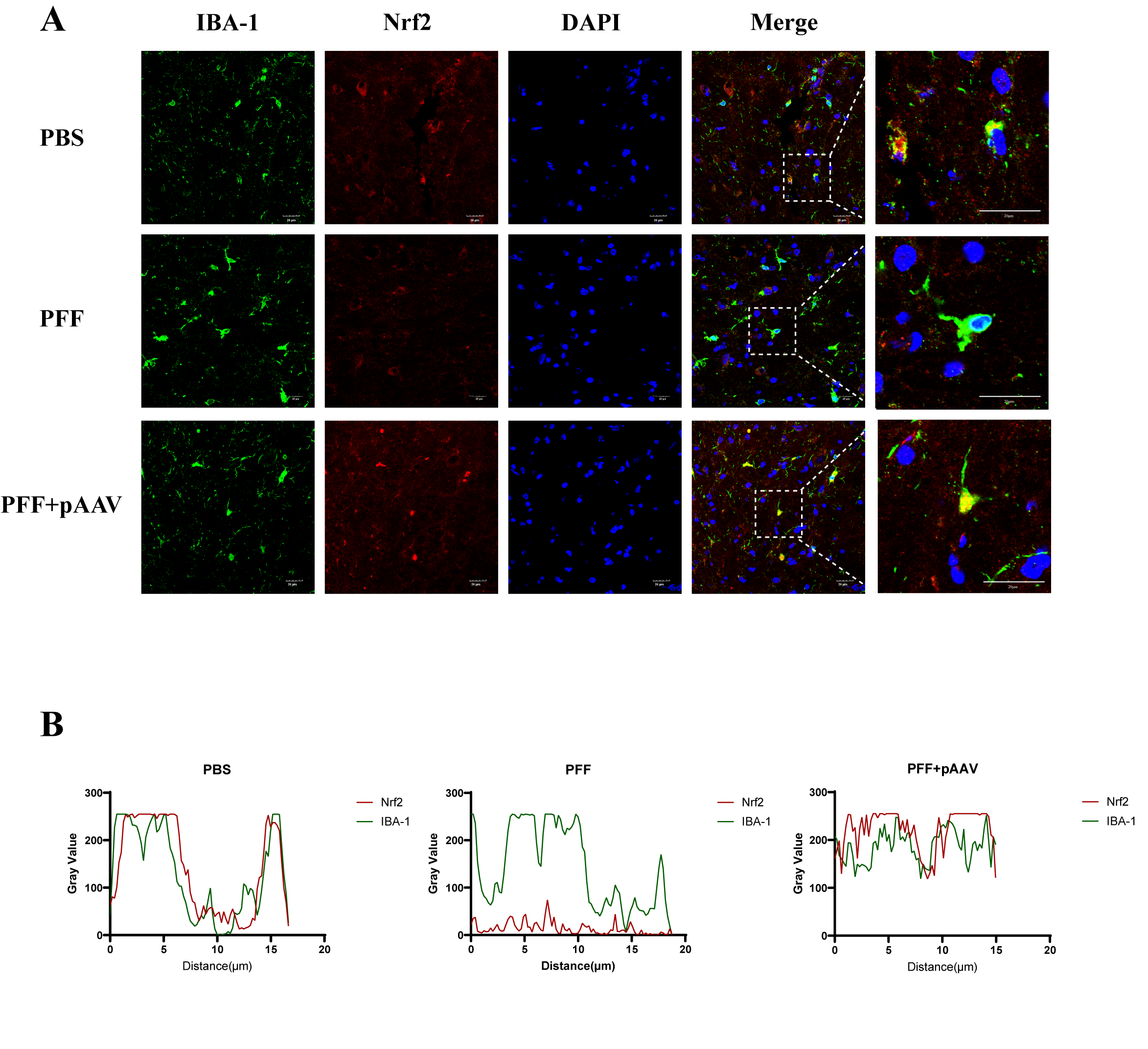

Supplement: Supplementary file 1 [file ijms-27-04579-s001.zip › ijms-4279756 - supplementray/Supplementary figure 1.tif]

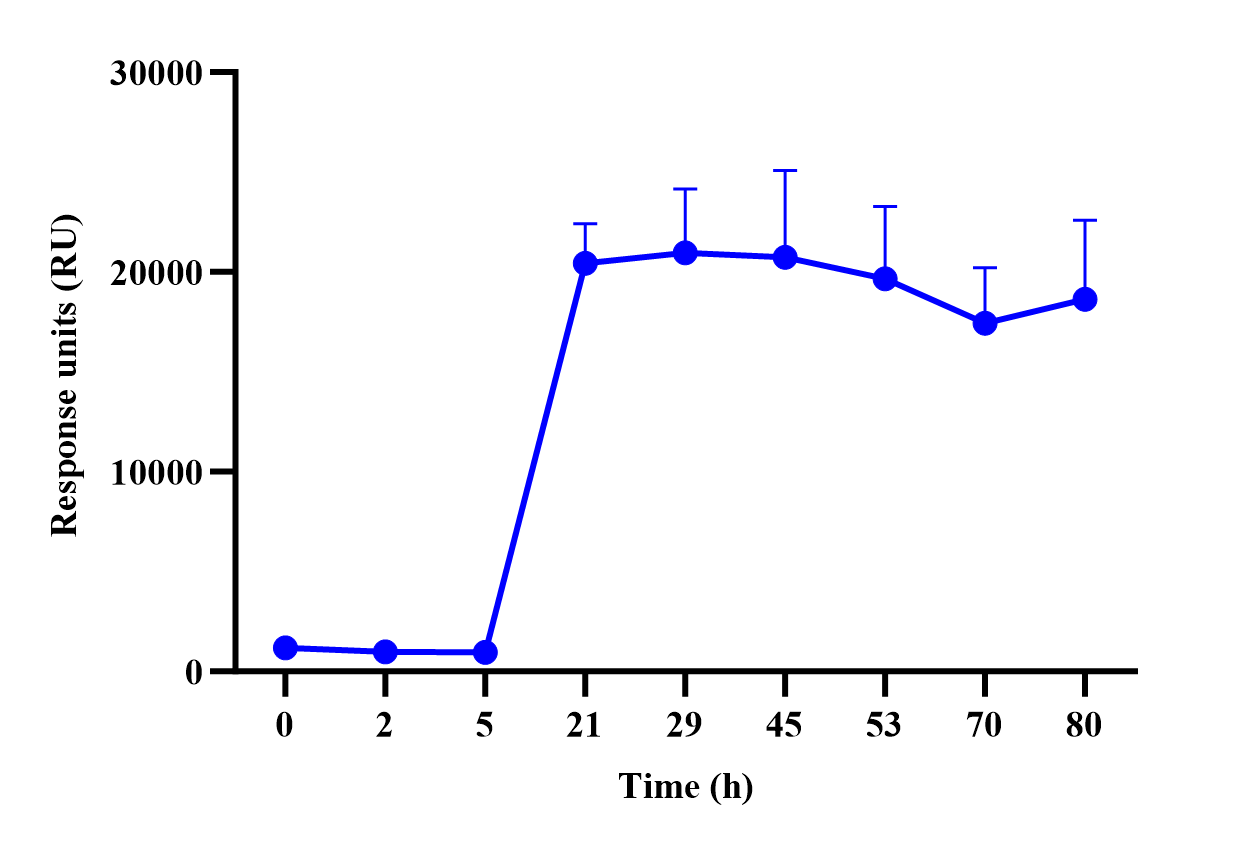

Supplement: Supplementary file 1 [file ijms-27-04579-s001.zip › ijms-4279756 - supplementray/Supplementary figure 2.tif]
